# Clinical manifestations and EEG findings in children infected with COVID-19 and exhibiting neurological symptoms

**DOI:** 10.1186/s12887-023-04496-y

**Published:** 2024-01-16

**Authors:** Yue Yang, Tao Yu, Jie Yang, Jia Luo, Xuan Liu, Chong Mu, Xiaochuan Wang, Yao Deng, Rong Luo

**Affiliations:** 1 Department of Pediatrics, West China Second University Hospital, Sichuan University, Chengdu, 610041 China; 2 Key Laboratory of Obstetric & Gynecologic and Pediatric Diseases and Birth Defects of Ministry of Education, Sichuan University, Chengdu, 610041 China

**Keywords:** COVID-19, EEG, Pediatrics, Seizures

## Abstract

Severe acute respiratory syndrome coronavirus 2 (SARS-CoV2) infection has many neurological manifestations, and its effects on the nervous system are increasingly recognized. There has been no systematic analysis of electroencephalography (EEG) characteristics in children exhibiting neurological symptoms of Coronavirus disease 2019 (COVID-19). The primary aim of this study was to describe the EEG characteristics caused by COVID-19 infection in children who were showing neurological symptoms and to assess the relationship between COVID-19-related EEG changes and clinical features in these children. Method: This study included 125 pediatric patients infected with SARS-CoV2 and showing neurological symptoms, and their continuous EEG was recorded. In addition, the demographic and clinical characteristics of these patients were analyzed and the correlation between the two was investigated. Results: Abnormal EEG findings were detected in 31.20% (N = 39) of the patients. Abnormal discharges (43.59%) were the most common EEG abnormalities, followed by background abnormalities (41.03%). The proportion of patients diagnosed with febrile seizure was higher in the normal EEG group than in the abnormal EEG group (*P* = 0.002), while the opposite was true for epilepsy and encephalitis/encephalopathy (*P* = 0.016 and *P* = 0.003, respectively). The independent associated factors of abnormal EEG were age and total length of stay (*P* < 0.001 and *P* = 0.003, respectively). Non-specific EEG abnormalities were found in COVID-19-related encephalitis/encephalopathy. Conclusion: Our study corroborated that a small group of pediatric patients infected by COVID-19 and showing neurological symptoms may exhibit abnormal EEG. This study could help improve the understanding of clinical and EEG characteristics in children with COVID-19 and inform triage policies in other hospitals during the COVID-19 pandemic.

## Introduction

Coronavirus disease 2019 (COVID-19) is a novel infectious disease caused by 2019 novel coronavirus (2019- nCoV) or severe acute respiratory syndrome coronavirus 2 (SARS-CoV2) [1]. Moreover, it is one of the most severe epidemics in human history [2]. During the COVID-19 epidemic, it was widely recognized that the virus could cause severe respiratory infections, and many patients were critically ill and required intensive care. Although respiratory failure is the signature complication of this infection, there is evidence that it also involves multiple systems. In particular, neurosystem-related complications have become a concern for medical researchers [3, 4]. COVID-19 infection may affect the central nervous system to very different degrees. Patients with COVID-19 were observed to develop encephalopathy, clinical seizures, and subclinical seizures. Electroencephalography (EEG) is an essential neurological diagnostic technique widely used to diagnose such disorders and guide related treatment decisions [5, 6].

Several previous studies have observed the neurological effects of COVID-19 by using EEG. In a retrospective study, Pellinen et al. analyzed continuous EEG findings in 111 patients with COVID-19. They showed a high rate of non-specific EEG abnormalities, while seizures and epileptiform activity were less frequent in the EEG [6]. In parallel, another study's researchers examined continuous and routine EEG of 22 patients. They found a higher frequency of epileptiform anomalies on EEG of COVID-19 patients with encephalopathy compared to the control subjects, and electroencephalographic seizures were observed in the COVID-19 patients [7].

As time passes after the first outbreak of the COVID-19 pandemic, there are increasing reports of pediatric COVID-19-infected patients presenting with neurologically relevant symptoms. Luckily, current data suggests that children diagnosed with COVID-19 often have milder diseases than adults, and deaths have been sporadic [8]. A meta-analysis showed that the percentage of children infected by COVID-19 with non-specific neurological symptoms such as headache and myalgia was 16.7%, and the portion of specific neurological symptoms such as seizures and encephalopathy was 1% [9]. In another study involving 50 children with COVID-19 infection, 4 (14.8%) patients presented with neurological symptoms, including encephalopathy, brainstem involvement with dysarthria or dysphagia, meningism, and cerebellar ataxia. EEG recordings showed diffuse slowing in 2 of these patients, while 1 had mild slow activity in the anterior part of the brain [10]. Another study reported a child with COVID-19 infection whose initial symptom was a headache, and her EEG showed remarkable background slowing and frequent frontal intermittent rhythmic discharges [11]. The current studies on the EEG performance of pediatric patients with COVID-19 are mainly case studies, and no studies have been conducted to analyze it systematically. Therefore, the presence or absence of specific EEG features for acute COVID-19 infection in children showing neurological symptoms has not been determined.

To the best knowledge of the authors of this study, this is the first study to date to investigate the clinical and EEG features in pediatric COVID-19-infected patients who show neurological symptoms. The main objectives of this study were to investigate the EEG manifestations caused by COVID-19 infection in children who were showing neurological symptoms and to assess the relationship between COVID-19-related EEG changes and clinical features in these children.

## Methods

### Study population and EEG recording

We performed a retrospective analysis at the Second West China Hospital of Sichuan University from December 1, 2022, to March 31, 2023 (COVID-19 pandemic in China). The study was divided into three stages. In the first stage, the inclusion criteria of subjects were: (1) age < 18; (2) the diagnosis of COVID-19 was confirmed by nasopharyngeal swab polymerase chain reaction (PCR); (3) patients were performed EEG because of neurological symptoms (e.g., suspected seizures or altered mental status). The exclusion criteria were: (1) patients with electrolyte or metabolic disorders; (2) patient was infected with common viruses or bacteria other than COVID-19, such as influenza virus, parainfluenza virus, adenovirus, respiratory syncytial virus, cytomegalovirus, mycoplasma, chlamydia, streptococcus pneumonia, haemophilus influenzae (throat swab specimens by PCR); (3) parents of the children refused to perform an EEG. In addition, to directly observe the effect of COVID-19 on EEG, we performed a second stage analysis. Patients included in the first stage who had previous EEG (before the infection of COVID-19) findings were included in the second stage of the study. Finally, to assess the EEG characteristics of COVID-19-related encephalitis or encephalopathy, the patients with encephalitis or encephalopathy from stage one were included in the analysis of stage three. All patients in this study underwent standard, continuous video and EEG monitoring using a 19-channel EEG with electrodes placed using the international 10–20 system for 4 h. This study was approved by the Ethics Committee of West China Second University Hospital, Sichuan University.

### Clinical variables

We extracted the data from the electronic medical record system. The extracted data included baseline demographic and clinical information: gender, age, fever (during the current episode), state of consciousness and Glasgow Coma Scale score at the time of performing the EEG, brain imaging findings (if any), diagnosis, clinical seizure before the EEG, form of seizure, presence of status epilepticus, seizure frequency compared to previous (if a patient's seizure interval was significantly shorter after infection with COVID-19 than before, then we considered that the patient's seizure frequency was increased, and vice versa), history of neurological illness, prior family history of seizures, whether take antiseizure medicine before EEG, and the interval time between EEG and last seizure.

In addition, we recorded whether the patient was hospitalized, the department where the patients stay (outpatient, neurology department, intensive care unit [ICU], infection department, transition ward, or other departments), the total length of stay, and the outcome at discharge as of March 31, 2023.

### EEG variables

EEG variables were abstracted from hospital EEG reports. Our EEG reporting physicians, who were qualified in EEG, read the EEG results and classified EEG according to the International League Against Epilepsy (ILAE) criteria. The EEG findings were categorized into normal and abnormal in the first stage (Tables 1 and 2). The abnormal EEG was further categorized into background abnormalities (background slowing and focal slowing), abnormal discharges, and background abnormalities combined with abnormal discharges. In the second stage (Table 3), the last EEG findings, EEG findings after infection with COVID-19, and the differences between these two EEGs were recorded. In addition, the details of background waves and abnormal discharges in patients with encephalitis/encephalopathy were recorded in the third stage (Table 4).

### Statistical methods

Clinical and EEG characteristics were summarized using descriptive statistics (mean and standard deviation of age, median [inter-quartile range, IQR] of other continuous variables, and proportion of categorical variables). To determine which factors of patients were most likely to be associated with abnormal EEG findings, we compared the normal and abnormal EEG groups’ demographic and clinical characteristics using univariate statistics (Mann–Whitney *U* test for continuous variables and chi-square for categorical variables). Probability (*P*) values of < 0.05 indicated statistical significance. Demographic and clinical variables with *P* < 0.1 were included in logistic regression analyses to assess the association between demographic and clinical characteristics and abnormal EEG. SPSS 22.0 (Statistical Package for Social Sciences for Windows, version 22.0.) software package was used to conduct the statistical analyses of the research data.

## Results

### Patient characteristics

Table 1 exhibits the demographic, clinical, and EEG characteristics of pediatric COVID-19 patients with neurological symptoms. Of the 125 pediatric patients, 77 (61.60%) were male, and 48 (38.40%) were female. The age of all patients ranged from 0.1 to 14.6 years, with a mean of 5.24 ± 3.44 years, of which 104 (83.20%) had a fever (during the current course). At the time of the EEG, most patients (*N* = 116, 92.80%) had a normal state of consciousness, and 9 (7.20%) patients had an abnormal state of consciousness. Four patients were rated by the Glasgow Coma Scale, with a median of 10.5 (7, 8, 13, and 15, respectively). Fifty-one patients had brain imaging studies completed, of which 11 (21.57%) were suggestive of abnormalities.

**Table 1:** Demographic, clinical and EEG characteristics

Variable						
Gender	Male			77	(61.60%)	
	Female			48	(38.40%)	
Age (Mean ± SD, year)				5.24 ± 3.44 ( overall range: 0.1–14.6)		
Fever	No			21	(16.80%)	
	Yes			104	(83.20%)	
Consciousness	Normal			116	(92.80%)	
	Abnormal			9	(7.20%)	
Glasgow Coma Scale score				10.5	(7.25,14.5)	*N* = 4
Brain image	Normal			40	(78.43%)	*N* = 51
	Abnormal			11	(21.57%)	*N* = 51
Diagnosis	Epilepsy			23^a^	(18.40%)	
	Febrile seizures			51	(40.80%)	
	Complex febrile seizures			29	(23.20%)	
	Febrile seizures plus			4	(3.20%)	
	Epileptic seizures			7	(5.60%)	
	Encephalitis^b^/encephalopathy			4	(3.20%)	
	Others			7	(5.60%)	
Clinical seizure	None			3	(2.40%)	
	Once			81	(64.80%)	
	Twice			19	(15.20%)	
	More than twice			22	(17.60%)	
Form of seizure	Generalized clinical			112	(91.80%)	*N* = 122
	Focal clinical			10	(8.20%)	*N* = 122
Presence of status epilepticus				6	(4.80%)	
Seizure frequency compared to previous	No prior seizures			66	(52.80%)	
	Increase			16	(12.80%)	
	Same as before			25	(20.00%)	
	Decrease			0	(0.00%)	
	Unknown			18	(14.40%)	
History of neurological illness	None			68	(54.40%)	
	Febrile seizures			35	(28.00%)	
	Epilepsy			15	(12.00%)	
	Others			7	(5.60%)	
Prior family history of seizures	None			115	(92.00%)	
	Yes			10	(8.00%)	
Antiseizure medicine before EEG	None			100	(80.00%)	
	Yes			25	(20.00%)	
Interval time between EEG and last seizure (median [IQR], day)				3	(1,8)	
Hospitalization	None			98	(78.40%)	
	Yes			27	(21.60%)	
Department	Outpatient			98	(78.40%)	
	Neurology department			15	(12.00%)	
	ICU			4	(3.20%)	
	Infection department			1	(0.80%)	
	Transition ward			3	(2.40%)	
	Other departments			4	(3.20%)	
Total length of stay (median [IQR], day)				7	(5,11)	
Outcome at discharge	Recovery			122	(97.60%)	
	Neurological sequalae			3	(2.40%)	
EEG	Normal			86	(68.80%)	
	Abnormal			39	(31.20%)	
		Background abnormalities		16	(41.03%)	*N* = 39
		Background slowing		11	(28.21%)	*N* = 39
		Focal slowing		5	(12.82%)	*N* = 39
		Abnormal discharges		17	(43.59%)	*N* = 39
		Background abnormalities + abnormal discharges		6	(15.38%)	*N* = 39

*Abbreviations*: *SD* standard deviation, *IQR* Interquartile range, *ICU* intensive care unit

^a^. Seven of the 23 patients were first diagnosed with epilepsy during our study period

^b^. The criteria of encephalitis were used as described in the Consensus Statement of the International Encephalitis Consortium [12]

The most frequent diagnosis was febrile seizures (*N* = 51, 40.80%); 29 (23.20%) patients were diagnosed with complex febrile seizures, 23 (18.40%) with epilepsy, and 4 (3.20%) with encephalitis [12]/encephalopathy, while the remaining diagnoses included: epileptic seizures (*N* = 7, 5.60%), febrile seizure plus (*N* = 4, 3.20%), and other diagnoses (*N* = 7, 5.60%). Almost all patients (*N* = 122, 97.60%) had seizures, and most (*N* = 81, 64.80%) had one seizure. The form of seizures was predominantly generalized clinical seizure (*N* = 112, 91.80%), and status epilepticus occurred in 6 patients (4.80%). Sixty-six patients (52.80%) had no previous history of seizures, 16 patients (12.80%) had an increase in current seizure frequency compared to previous (seven epilepsy, six complex febrile seizures, two febrile seizures plus, and one febrile seizure), and 25 patients (20.00%) had no change in seizure frequency.

Most of the patients (*N* = 68, 54.40%) had no prior history of neurological diseases. Thirty-five patients (28.00%) had a prior history of febrile seizure, and 15 patients (12.00%) had a prior history of epilepsy. Most patients (*N* = 115, 92.00%) had no prior family history of seizures, while ten patients (8.00%) had a prior family history of seizures.

Most patients (*N* = 100, 80.00%) did not take antiseizure medicine before EEG, and 25 (20.00%) patients took such medicine, including levetiracetam (*N* = 12), valproic acid (*N* = 8), oxcarbazepine (*N* = 4), phenobarbital (*N* = 4), lamotrigine (*N* = 3), topiramate (*N* = 2), clonazepam (*N* = 2) and lacosamide (*N* = 1). Eight of the patients were taking two kinds of medicines at the same time, and three patients were taking three kinds of the medicines. The median length of interval time between an EEG performed and the last seizure was three days (IQR = 1 to 8 days).

Twenty-seven patients (21.60%) were hospitalized in the neurology department (*N* = 15, 12.00%), ICU (*N* = 4, 3.20%), infection department (*N* = 1, 0.80%), transition ward (*N* = 3, 2.40%), and other departments (*N* = 4, 3.20%). The median length in the hospital was seven days (IQR = 5 to 11 days). One hundred and twenty-two patients recovered at the time of discharge, and three patients (2.40%) were discharged with neurological sequelae, one with a language barrier, one with dystonia, and the remaining one with both language barrier and dystonia.

### EEG findings

The 125 patients with COVID-19 in this study all had one EEG during this course of the disease. Most patients (68.80%) had normal EEG, and 39 patients (31.20%) had abnormal EEG. Of these, abnormal discharges were the most common (*N* = 17, 43.59%). The EEGs of sixteen patients (41.03%) exhibited background abnormalities, and it could be further divided into background slowing (28.21%) as well as focal slowing (12.81%). The EEGs of six patients (15.38%) exhibited background abnormalities + abnormal discharges.

### Difference of normal and abnormal EEG

Eighty-six patients with normal EEG and 39 patients with abnormal EEG were included in this study (Table 2). The rate of males in the normal EEG group was higher than in the abnormal EEG (*P* = 0.046), and the age of patients in the normal EEG group was significantly younger than that of patients in the abnormal EEG group (*P* < 0.001). Abnormal EEG was associated with the level of consciousness. Patients in the normal EEG group were more likely to be conscious (*P* = 0.017). The proportion of patients diagnosed with febrile seizures (*P* = 0.002) was higher in the normal EEG group than in the abnormal EEG group, while the opposite was true for epilepsy and encephalitis/encephalopathy (*P* = 0.016 and *P* = 0.003, respectively). The form of the seizure (*P* = 0.006) and history of neurological illness (febrile seizures *P* = 0.034) were significantly different between the two groups. The rate of antiseizure medicine used before EEG was higher in the abnormal EEG group than in the normal EEG group (*P* = 0.001), and the interval time between an EEG performed and the last seizure was significantly longer in the normal EEG group than in the abnormal EEG group (*P* = 0.001). In addition, patients with normal EEG had a lower hospitalization rate, shorter stay, and higher recovery rate (*P* = 0.002,* P* < 0.001, and *P* = 0.009, respectively). There was no statistically significant difference between the fever rate, brain image, diagnosis (epilepsy, complex febrile seizures, febrile seizures plus, and epileptic seizures), clinical seizure, status epilepticus, increased frequency of seizures, history of epilepsy, and prior family history of seizures.

**Table 2:** Difference between the groups in terms of different parameters

Variable			Normal EEG			Abnormal EEG			*P*
Gender	Male		58	(67.44%)		19	(48.72%)		0.046
	Female		28	(32.56%)		20	(51.28%)		
Age ( Mean ± SD, year)			4.38 ± 3.10			7.11 ± 3.44			< 0.001
Fever			75	(87.21%)		29	(74.36%)		0.075
Consciousness	Normal		83	(96.51%)		33	(84.62%)		0.017
	Abnormal		3	(3.49%)		6	(15.38%)		
Brain image	Normal		25	(80.65%)	*N* = 31	15	(75.00%)	*N* = 20	0.632
	Abnormal		6	(19.35%)	*N* = 31	5	(25.00%)	*N* = 20	
Diagnosis	Epilepsy	No	75	(87.21%)		27	(69.23%)		0.016
		Yes	11	(12.79%)		12	(30.77%)		
	Febrile seizures	No	43	(50.00%)		31	(79.49%)		0.002
		Yes	43	(50.00%)		8	(20.51%)		
	Complex febrile seizures	No	64	(74.42%)		32	(82.05%)		0.349
		Yes	22	(25.58%)		7	(17.95%)		
	Febrile seizures plus	No	83	(96.51%)		38	(97.44%)		0.786
		Yes	3	(3.49%)		1	(2.56%)		
	Epileptic seizures	No	82	(95.35%)		36	(92.31%)		0.493
		Yes	4	(4.65%)		3	(7.69%)		
	Encephalitis/encephalopathy	No	86	(100.00%)		35	(89.74%)		0.003
		Yes	0	(0.00%)		4	(10.26%)		
Clinical seizure			84	(97.67%)		38	(97.44%)		0.936
Form of seizure	Generalized clinical		81	(96.43%)	*N* = 84	31	(81.58%)	*N* = 38	0.006
	Focal clinical		3	(3.57%)	*N* = 84	7	(18.42%)	*N* = 38	
Presence of status epilepticus			3	(3.49%)		3	(7.69%)		0.308
Increased frequency of seizures			11	(28.95%)	*N* = 38	5	(23.81%)	*N* = 21	0.535
History of neurological illness	Epilepsy	No	80	(93.02%)		30	(76.92%)		0.1
		Yes	6	(6.98%)		9	(23.08%)		
	Febrile seizures	No	57	(66.28%)		33	(84.62%)		0.034
		Yes	29	(33.72%)		6	(15.38%)		
Prior family history of seizures			8	(9.30%)		2	(5.13%)		0.425
Antiseizure medicine before EEG			10	(11.63%)		15	(38.46%)		0.001
Interval time between EEG and last seizure (median [IQR], day)			4	(2,8)		2	(1,8)		0.001
Hospitalization			12	(13.95%)		15	(38.46%)		0.002
Total length of stay (median [IQR], day)			6	(4,8)		9	(6,20)		< 0.001
Outcome at discharge	Recovery		86	(100.00%)		36	(92.31%)		0.009
	Neurological sequalae		0	(0.00%)		3	(7.69%)		

*Abbreviations*: *SD* standard deviation, *IQR* Interquartile range

### Independent associated factors of abnormal EEG

Among clinical variables, including demographics, medical or neurological manifestation, medications, and hospitalization, the independent associated factors of abnormal EEG were age (odds ratio [OR] 1.295, 95% confidence interval [CI] 1.134–1.478, *P* < 0.001) and the total length of stay (OR 1.212, 95% CI 1.065–1.380, *P* = 0.003).

### Comparison of previous and current EEGs

As shown in Table 3, 11 children with previous EEG were included in this study, suffering from epilepsy, febrile seizures, and complex febrile seizures. Ten patients exhibited generalized clinical seizures, and one exhibited focal clinical seizure. Three children with epilepsy were taking antiseizure medicine. Six children infected with COVID-19 had varying degrees of severity of background wave slowing or slow waves. Compared with the previous EEG, three patients with COVID-19 infection exhibited slower background waves, three affected patients showed discharge range expansion, and one patient exhibited increased discharge.

**Table 3:** Comparison of previous and current EEGs

Number	Gender	Age (years)	Diagnosis	Presence of status epilepticus	Fever	Number of seizures	Form of seizure	History of neurological illness	Antiseizure medicine before EEG	Seizure frequency compared to before	Age of last EEG (year)	Outcomes of last EEG	EEG findings after infection with COVID-19	Differences between the two EEGs
1	Male	14.6	Epilepsy	No	Yes	1	Generalized clinical	Epilepsy	PB, VPA and TPM	Unknown	14.3	Waking sleep period prefrontal, frontal, central, parietal, occipital, temporal, midline areas or widespread spikes or spines (slow), multi-spine slow, slow wave frequency (with widespread predominance and NREM period discharge index up to 50%)	Background wave slowing	Background slowing (6 Hz), while discharge reduced
2	Male	10.6	Epilepsy	No	No	1	Generalized clinical	Epilepsy	No	Same as before	9.1	Central, parietal, and middle and posterior temporal regions with frequent issuance of low spike, spine, and multi-spine waves	Frequent spikes and slow waves in the frontal, central, parietal, occipital and temporal regions or widespread spikes and slow waves during sleep (predominantly in the right central region)	Discharge range expansion
3	Male	6.6	Epilepsy	No	Yes	2	Generalized clinical	Epilepsy	No	Increase	5.5	Several posterior temporal spike waves issued during sleep period	1. slight slowing of background waves; 2. frequent emission of (multiple) spines (slow) and slow waves in the prefrontal and frontal regions during waking and sleeping periods	Background slowing (8–9 Hz [5 years 6 months] → 6–8 Hz [6 years 7 months])
4	Male	5.5	Epilepsy	No	Yes	1	Focal clinical	Epilepsy	VPA	Same as before	4.4	Abnormal preschool EEG: multiple emission of sharp and spike waves in the right central region during sleep	Occasional release of left frontal and central zone sharp waves during sleep	Discharge range expansion
5	Male	3.3	Epilepsy	No	No	1	Generalized clinical	Epilepsy	VPA and LCM	Same as before	2.6	Normal	Normal	None
6	Female	4.6	Febrile seizures	No	Yes	1	Generalized clinical	Febrile seizures	No	Same as before	3.1	No abnormalities were observed	1. multiple issuances of slow and slow waves in the prefrontal, frontal, central, parietal, occipital, posterior temporal regions or widespread spikes (spines) during waking sleep (with widespread predominance); 2. positive photo paroxysm response	Increased discharges
7	Female	3.7	Febrile seizures	No	Yes	1	Generalized clinical	Febrile seizures	No	Same as before	1.6	Occasional issuance of frontal spikes with background waves of 4–6 Hz	1. slight slowing of background waves; 2. frequent issuance of δ and θ slow waves in the occipital and posterior temporal regions during waking and sleeping periods	Background slowing (4–6 Hz [1 year 7 months] → 4–8 Hz [3 years 7 months]), predominantly θ waves
8	Male	3.3	Febrile seizures	No	Yes	1	Generalized clinical	Febrile seizures	No	Same as before	1.4	Occasional spike waves in the frontal area during sleep	Occasional release of sharp waves in the frontal, frontal midline, and central midline regions during sleep	Discharge range expansion
9	Male	10.7	Complex febrile seizures	No	Yes	1	Generalized clinical	Febrile seizures	No	Increase	8.7	No abnormalities were observed	Closed-eye sensitivity	Presence of closed-eye sensitivity
10	Male	7.8	Complex febrile seizures	No	Yes	1	Generalized clinical	Complex febrile seizures	LEV	Same as before	7.4	Normal range EEG	Normal range EEG	None
11	Male	1.9	Complex febrile seizures	Yes	Yes	2	Generalized clinical	Complex febrile seizures, Delayed language development	No	No prior seizures	1.7	No abnormalities were observed	1. multiple δ-slow waves in the prefrontal, frontal, and frontal midline regions are seen during the waking period; 2. several sharp waves in the prefrontal and frontal regions are seen during the sleeping period	Presence of focal slow waves

*Abbreviations:* *PB* phenobarbital, *VPA* valproic acid, *TPM* Topiramate, *LCM* Lacosamide, *LEV* levetiracetam

### Detailed EEG in patients with encephalitis/encephalopathy

**Fig. 1:**
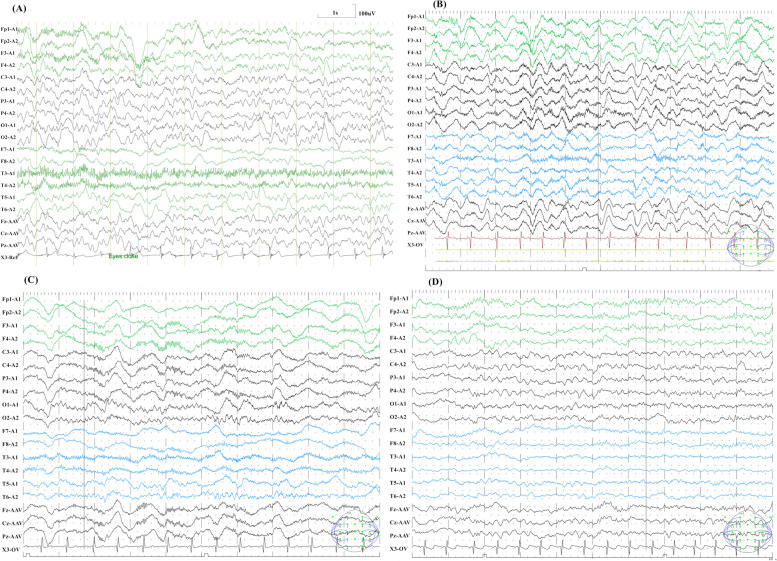
EEGs of patients suffered from encephalitis/encephalopathy. All patients showed different degrees of EEG background wave slowing. **A** was from patient 1 and showed diffuse δ-θ slowing waves. **B** Was from patient 2 and showed diffuse δ slowing waves. Figure (**C**) was from patient 3 and showed diffuse δ slowing waves with a few α rhythms in the occipital region. Figure (**D**) was from patient 4 and showed diffuse θ slowing waves with a few δ slowing waves

**Fig. 2:**
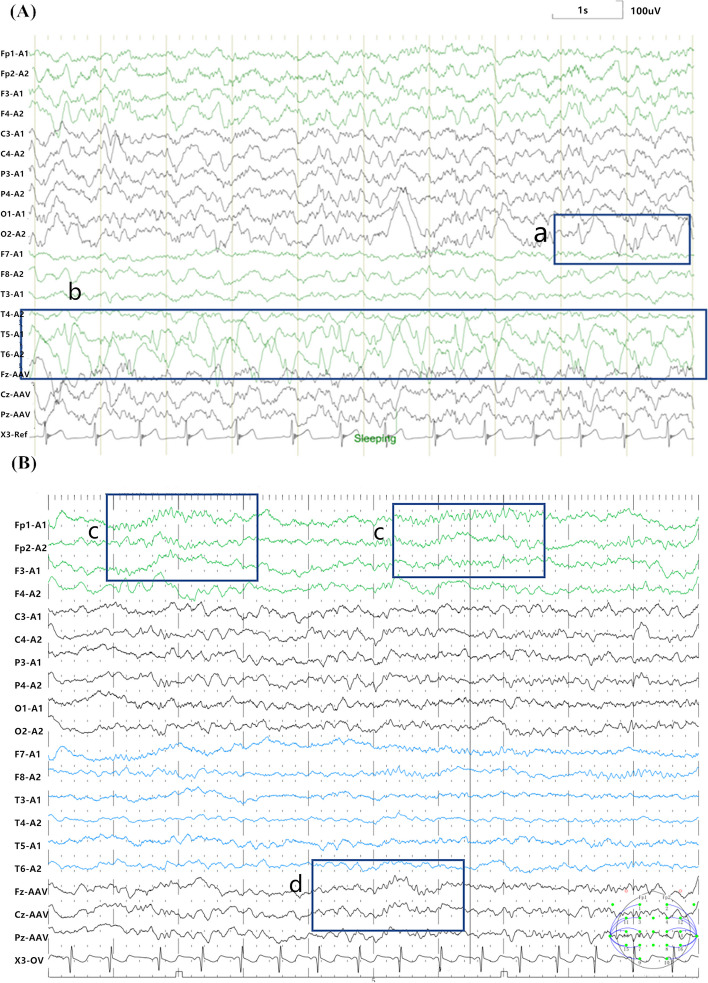
Abnormal discharges of patients suffered from encephalitis/encephalopathy. **A** From Patient 1, the EEG showed sharp and slow wave discharges in the patient's (**a**) right occipital region and (**b**) left and right posterior temporal regions. **B** From Patient 4, EEG showed low amplitude α rhythmic discharges in the patient's (**c**) bilateral prefrontal, frontal, and (**d**) frontal midline regions

A total of 4 patients in this study suffered from encephalitis/encephalopathy. As shown in Fig. 1, all patients showed different degrees of EEG background wave slowing, and one of the patients with encephalopathy also exhibited typical sleep spindle and parietal wave deficits, and unclassifiable sleep cycles. In addition, the EEGs of 2 patients showed abnormal discharges (Fig. 2). However, these findings were non-specific EEG abnormalities. Three patients were discharged with neurologic sequelae, including language barrier and dystonia, details of which are shown in Table 4.

**Table 4:** Detailed EEG in patients with encephalitis/encephalopathy

Number	Gender	Age (year)	Diagnosis	Presence of status epilepticus	Fever	Number of seizures	Form of seizure	History of neurological illness	Antiseizure medicine before EEG	Background waves	Abnormal discharges	Neurologic sequelae at discharge
1	Female	8.8	Viral encephalitis	None	Yes	7	Focal clinical	None	PB	4-6 Hz	Frequent occipital and posterior temporal sharp (slow) and slow waves during waking and sleeping periods (notably during sleep, predominantly in the right posterior temporal region)	None
2	Female	2.3	Severe viral encephalitis	Yes	Yes	7	Generalized clinical	None	PB、LEV	1.5-3 Hz	None	Language barrier and dystonia: there was no verbal response and low muscle tone in the extremities
3	Male	8.7	Anti-N-methyl-D-aspartate receptor encephalitis	None	Yes	1	Focal clinical	None	None	2.5-7 Hz	None	Language barrier: language nonsense
4	Female	11.6	Acute encephalopathy syndrome	None	Yes	1	Generalized clinical	None	None	1.5-9 Hz; Typical sleep spindle and top wave deficits, with unclassifiable sleep cycles	Multiple discharges of α and θ rhythms in the prefrontal, frontal, central, anterior temporal regions or in the full leads during wakefulness and sleep	Dystonia: slightly high muscle tone in the right upper limb

*Abbreviations*: *PB* phenobarbital, *LEV* levetiracetam

## Discussion

As the COVID-19 pandemic continues, there is a need to better understand the neurological manifestations and EEG features of COVID-19. This study reports the first large series of clinical and EEG characteristics associated with pediatric COVID-19 infections. The prevalence of abnormal EEG was 31.20%, and these abnormal EEG findings increased significantly with age and total length of stay. Abnormal discharges (43.59%) were the most common EEG abnormalities, followed by background abnormalities (41.03%). The proportion of patients diagnosed with febrile seizures was higher in the normal EEG group than in the abnormal EEG group, while the opposite was true for epilepsy and encephalitis/encephalopathy. Our study is the first to specifically examine the effect of various demographic and clinical variables on the EEG results of pediatrics who showed neurological symptoms infected by COVID-19 to strengthen or refute the existing practice. The information in this article may be important in guiding critical clinical decisions to treat this disease.

Our study's clinical presentations and demographic pattern (including male predominance) related to COVID-19 infections were similar to a previous survey researched in an Italian Pediatric Center [13]. The proportions of clinical seizures in patients with COVID-19 have been reported to be 0.08–0.5% [14, 15]. A recent study suggests seizures may be the initial manifestation of SARS-CoV-2 infection in children, in the absence of a prior history of neurological disorders, and a prevalence of about 18% of seizures in pediatric patients with COVID-19 [13]. Our study found that febrile seizures were the most common illness in this study population. Febrile seizures are the most common seizure in childhood, with an incidence of 2 to 5% [16]. Most patients (83.20%) in our study had a fever. The febrile response in patients with febrile seizures could also reflect an altered function of the cytokine network, with IL-1 and IL-6 being the most likely involved mediators [17]. On the other hand, the SARS-CoV-2 virus induces a systemic inflammatory response, which may promote increased cytokine release, leading to the onset of febrile seizures [18]. In three patients with febrile convulsions who had previous EEG comparisons, we found that all EEGs showed more severity after infection with COVID-19, as evidenced by background slowing, discharge range expansion, and abnormal discharges. However, the severity of the EEGs in these cases was mild, and the EEGs were performed during fever, so we could not know whether the abnormal EEG changes were due to fever or to direct viral invasion of the nervous system.

Acute symptomatic epileptic seizures and status epilepticus are two of the most frequently reported neurological conditions associated with SARS-CoV-2 infection and carry a high mortality rate (between 3 and 50%) [19]. Status epilepticus was reported in 4.5% of the patients [20], comparable to 4.8% in our study. A recent systematic review suggested that patients with pre-existing neurological disorders (including epilepsy) may develop worsening neurological problems after being infected with COVID-19 [21]. This finding was also confirmed in our study, twenty-three patients included in this study suffered from epilepsy, and seven had more frequent seizures after COVID-19 infection than before. In addition, some adult reports have speculated that up to 3% of patients with severe COVID-19 illness have subclinical seizures [22]. However, in children, it has not been explored.

A previous systematic review of 177 patients reported nonspecific EEG findings in patients with COVID-19 [20]. Our study showed that most patients had normal EEG, and 31.20% had abnormal EEG. Part of the abnormal EEG recordings showed nonspecific EEG abnormalities of background rhythm, like generalized/ focal slowing and abnormal discharges. This result suggests that pediatric patients infected with COVID-19 and showing neurological symptoms may likely have encephalopathy. Clinical seizure, fever, and antiseizure medicine may cause encephalopathy. COVID-19 can also directly invade the central nervous system or cause encephalopathy through inflammatory responses mediated by cytokine storms [23]. In addition, a small number of patients showed focal EEG abnormalities, consistent with previous research [24]. Focal abnormalities might be caused by complications of COVID-19, for example, encephalitis [25]. Moreover, the patient’s past medical history of brain disease or preexisting chronic neurological diseases may also affect focal findings on the EEG. There were ten patients with epilepsy in the abnormal EEG, including nine patients with a previous diagnosis of epilepsy, which further indicates that the abnormal EEG cannot be ruled out as caused by the previous disease. In addition, based on the comparison of EEG before and after infection in epileptic patients, we found no significant worsening of the EEG. Therefore, we believe that COVID-19 did not necessarily lead to abnormalities in EEG. This speculation requires a large sample size and control group experiments for validation. Moreover, it is worth noting that the interval time between EEG and the last seizure in the abnormal EEG was significantly shorter than that in the normal EEG. This might be because EEG recording in the early period increases the probability of abnormality.

Another important finding in our study was the independent associated factors of abnormal EEG. The abnormal EEG findings increased significantly with age and total length of stay. The mean age of the abnormal patients was significantly higher than those with normal EEG. A study in adults also found that abnormal EEG increased with age [26], but the authors did not explain. As for our result, we speculated this was due to the fact that more patients at younger ages were diagnosed with febrile seizures. Febrile seizures usually appear in children between 1 and 5 years old, and the EEG is usually normal [27]. In addition, the total length of stay was more likely to be associated with abnormal EEG. This is easily explained by the fact that patients with abnormal EEGs tend to be more severely ill and, therefore, require hospitalization for a more extended period.

Detailed EEGs of four patients with encephalitis or encephalopathy were also analyzed in this study. It was found that the EEGs of these patients in this study were non-specific, with slow background activity. This result was consistent with previously reported EEG findings in two cases of encephalitis in COVID-19 pediatric patients [28, 29]. Moreover, we noted abnormal discharge in the frontal region of the patient suffering from acute encephalopathy syndrome. A systematic review also found this phenomenon and hypothesized that this manifestation correlated with the purported entry of COVID-19 into the brain and proposed a viral spread hypothesis [30]. The researchers suggested that the virus first entered the nasal and oral mucosa (anosmia and ageusia) [31], then spread to the orbitofrontal region [32] and invaded the olfactory bulb and orbitofrontal/frontal region via afferent nerves.

There were several limitations in this study. First of all, 20.0% of the patients included in this study had taken antiseizure medicine before the EEG, which would affect the results of the EEG, such as increasing the probability of normal EEG. Secondly, COVID-19 was only confirmed by nasopharyngeal swab PCR and the lack of tests for COVID-19 in the cerebrospinal fluid, which made it difficult to associate this virus with neurological symptoms. Finally, follow-up data after discharge were hard to obtain, so long-term functional outcomes could not be assessed in this study. In future studies, long-term follow-ups of patients infected with COVID-19 will be conducted, and outcomes after discharge will be compared to evaluate the extent of COVID-19-related impact on long-term neurological functioning.

## Conclusion

Our study corroborates that a small subgroup of pediatric patients infected by COVID-19 and showing neurological symptoms may exhibit abnormal EEGs, and age and total length of stay were associated with them. This study could help improve the understanding of clinical and EEG characteristics in children who showed neurological symptoms and were infected by COVID-19, and inform EEG triage policies in other hospitals during the COVID-19 pandemic. Future researches on the relationship of the EEG findings to the clinical state and short- or long-term prognosis of COVID-19 patients may be conducted to help clinicians discern which patients would necessitate an EEG procedure. It would eventually require treatment with the ultimate aim of improving their clinical outcomes.

## Data Availability

The data that support the findings of this study are available on request from the corresponding author. The data are not publicly available due to privacy or ethical restrictions.
